# Biological risk based on preoperative serum CA19‐9 and histological grade predicts prognosis and improves accuracy of classification in patients with pancreatic ductal adenocarcinoma

**DOI:** 10.1002/cnr2.1911

**Published:** 2023-10-12

**Authors:** Shaofei Chang, Yaohua Liu, Yuexiang Liang, Quan Man, Haorui Li, Yu Guo, Tiansuo Zhao

**Affiliations:** ^1^ Department of Pancreatic Cancer, Tianjin Medical University Cancer Institute and Hospital, National Clinical Research Center of Cancer, Key Laboratory of Cancer Prevention and Therapy Tianjin's Clinical Research Center For Cancer Tianjin China; ^2^ Department of Gastroenteropancreas Shanxi Provincial People's Hospital Taiyuan China; ^3^ Graduate School Shanxi Medical University Jinzhong China; ^4^ Department of Ultrasound The Second People's Hospital of Shanxi Province Taiyuan China

**Keywords:** CA19‐9, histological grade, pancreatic ductal adenocarcinoma, prognosis, TNM, tumor biology

## Abstract

**Background:**

Carbohydrate antigen (CA) 19‐9 and histological grade can serve as indicators of the biological characteristics of pancreatic ductal adenocarcinoma (PDAC).

**Aims:**

The aim of this study was to investigate the combined impact of preoperative CA19‐9 levels and histological grade on prognosis and classification accuracy in PDAC patients.

**Methods and results:**

A retrospective cohort study was conducted on 612 patients with PDAC who underwent curative pancreatectomy, and a biological risk model based on preoperative CA19‐9 levels and histology grade was established. The prognostic importance of the biological risk model was evaluated, and its validity was confirmed through a validation cohort of 218 patients. The survival of patients with PDAC was independently associated with preoperative CA19‐9 levels and histology grade, indicating a biological risk. This biological risk was incorporated into the eighth edition of the TNM staging system, leading to the development of a modified TNM (mTNM) staging system. Receiver operating characteristic (ROC) curves demonstrated that the mTNM staging system had a significantly larger area under the curve (AUC) than the TNM staging system. The discriminatory capacity of the mTNM staging system was further validated in an independent cohort.

**Conclusion:**

Biological risk based on preoperative CA19‐9 and histological grade could predict the survival of patients with PDAC. The incorporation of biological risk into the TNM staging system has the potential to enhance the accuracy of patient classification in PDAC, predicting patient survival and enabling the development of individualized treatment plans.

## 1 INTRODUCTION

Pancreatic ductal adenocarcinoma (PDAC) is one of the deadliest malignancies. According to the World Health Organization, the estimated number of new cases of pancreatic cancer ranked 12th among all malignant tumors in 2020.^1^ The 5‐year survival rate is approximately 11.5%.^2^

Multiple disciplinary therapy based on the tumor stage and biological characteristics of the primary lesion is highlighted as a means to improve the survival of patients with PDAC. For example, research has verified that patients with node‐negative (N0) pancreatic cancer may benefit from adjuvant therapy after neoadjuvant therapy and surgical resection. This survival benefit may be most pronounced in patients with perineural invasion.^3^

The American Joint Committee on Cancer (AJCC) tumor‐node‐metastasis (TNM) staging system is the most commonly used classification for prognostic evaluation and treatment decisions in PDAC (Table 1).^4^ The TNM classification has continuously improved to accurately reflect the current understanding of disease extent.^5^, ^6^ Several studies have clarified the accuracy of the TNM staging of pancreatic cancer (8th ed., 2017).^7^, ^8^, ^9^ In contrast, several other studies have demonstrated deficiencies in survival differences between substages of the TNM classification and proposed a modified TNM or a newly developed staging system.^10^, ^11^ Therefore, it may be necessary to combine the AJCC TNM stage with tumor biological characteristics to improve the classification of PDAC.

**TABLE 1:** AJCC prognostic groups.

	T	N	M
Stage 0	Tis	N0	M0
Stage IA	T1	N0	M0
Stage IB	T2	N0	M0
Stage IIA	T3	N0	M0
Stage IIB	T1,T2,T3	N1	M0
Stage III	T1,T2,T3	N2	M0
	T4	Any N	M0
Stage IV	Any T	Any N	M1

*Note*: T, primary tumor; T0, no evidence of primary tumor; Tis, Carcinoma in situ; T1, tumor ≤2 cm in greatest dimension; T2, tumor >2 cm and ≤4 cm in greatest dimension; T3, tumor >4 cm in greatest dimension; T4, Tumor involves the celliac axis, superior mesenteric artery, and/or common hepatic artery, regardless of size; N, regional lymph nodes; N0, no regional lymph node metastasis; N1, metastasis in one to three regional lymph nodes; N2, metastasis in four or more regional lymph nodes; M, distant metastasis; M0, no distant metastasis; M1, distant metastasis.

Tumor biology has a substantial impact on the prognosis of patients with malignancy and has been incorporated into the staging system to improve the accuracy of prognostic prediction.^12^, ^13^ Since both tumor burden and malignant degree could reflect the characteristics of tumor biology,^14^, ^15^ we hypothesize that combining factors representing tumor burden and malignant degree would provide a comprehensive understanding of tumor biology in predicting cancer prognosis. Incorporating factors associated with tumor biology into the staging system could enhance the accuracy of TNM staging in predicting prognosis. Carbohydrate antigen 19–9 (CA19‐9) is associated with tumor burden,^16^ and histology grade is associated with the degree of malignancy.^17^, ^18^ Therefore, the combination of preoperative serum CA19‐9 and histological grade can accurately reflect the tumor biology of PDAC. In the present study, we retrospectively analyzed the data of 612 patients with PDAC who underwent curative resections to explore the effect of tumor biology based on preoperative serum CA19‐9 levels and histology grade on prognosis and the accuracy of patient classification in PDAC.

## 2 MATERIALS AND METHODS

This study was approved by the Ethical Review Committees of Tianjin Medical University Cancer Institute and Hospital and was conducted in accordance with the ethical guidelines of the Declaration of Helsinki. Informed consent was waived due to the retrospective nature of the study.

### 2.1 Patients

This study was conducted using a training cohort and an independent validation cohort. The training cohort consisted of 612 patients with PDAC who underwent curative resection at the Department of Pancreatic Cancer, Tianjin Medical University Cancer Institute and Hospital between January 1, 2011, and December 31, 2018. As this study is a retrospective study, patients of any age were enrolled. Therefore, the new staging system is suitable for all patients regardless of their age range. All patients underwent either a partial or total pancreatectomy, with superior mesenteric vein or portal vein resection being performed in cases where vascular infiltration was suspected. Standard lymph node dissection was routinely carried out, excluding patients who accepted palliative surgery or exploratory laparotomy without resection as well as those with missing values of preoperative CA19‐9 and histology tumor grade. Therefore, the inclusion criteria for this study comprised (1) patients diagnosed with PDAC, (2) patients who underwent pancreatectomy with curative intent, (3) patients without distant metastasis, (4) patients with no history of other malignancies, (5) patients who were not lost to follow‐up and (6) those who did not die during their initial hospital stay or within one month postsurgery. The validation cohort included 218 patients from the First Affiliated Hospital of Hainan Medical University and Shanxi Provincial People's Hospital between January 1, 2015, and December 31, 2018, who met the same criteria.

### 2.2 Evaluation of clinicopathological variables and survival

The clinicopathological variables studied included 14 factors: sex, age at surgery, preoperative serum levels of CA19‐9, carcinoembryonic antigen (CEA), and CA242, tumor location, type of surgery, TNM stage, T stage, N stage, histology grade, lymphovascular invasion, perineural invasion and postoperative adjuvant chemotherapy.

Preoperative serum tumor markers (CA19‐9, CEA, and CA242) were detected within one week prior to surgery. The normal upper limits of serum tumor markers were adopted as follows: CA19‐9 (37.0 U/mL), CEA (5.0 ng/mL) and CA242 (20 U/mL). In this study, all patients with a total bilirubin level ≥ 250 mmol/L were treated with either percutaneous transhepatic biliary drainage or endoscopic retrograde biliary drainage. After biliary drainage for approximately 1–2 weeks, the operation was performed if the total bilirubin level was less than 100 mmol/L, and the preoperative serum CA19‐9 level was rechecked along with bilirubin.

**FIGURE 1:**
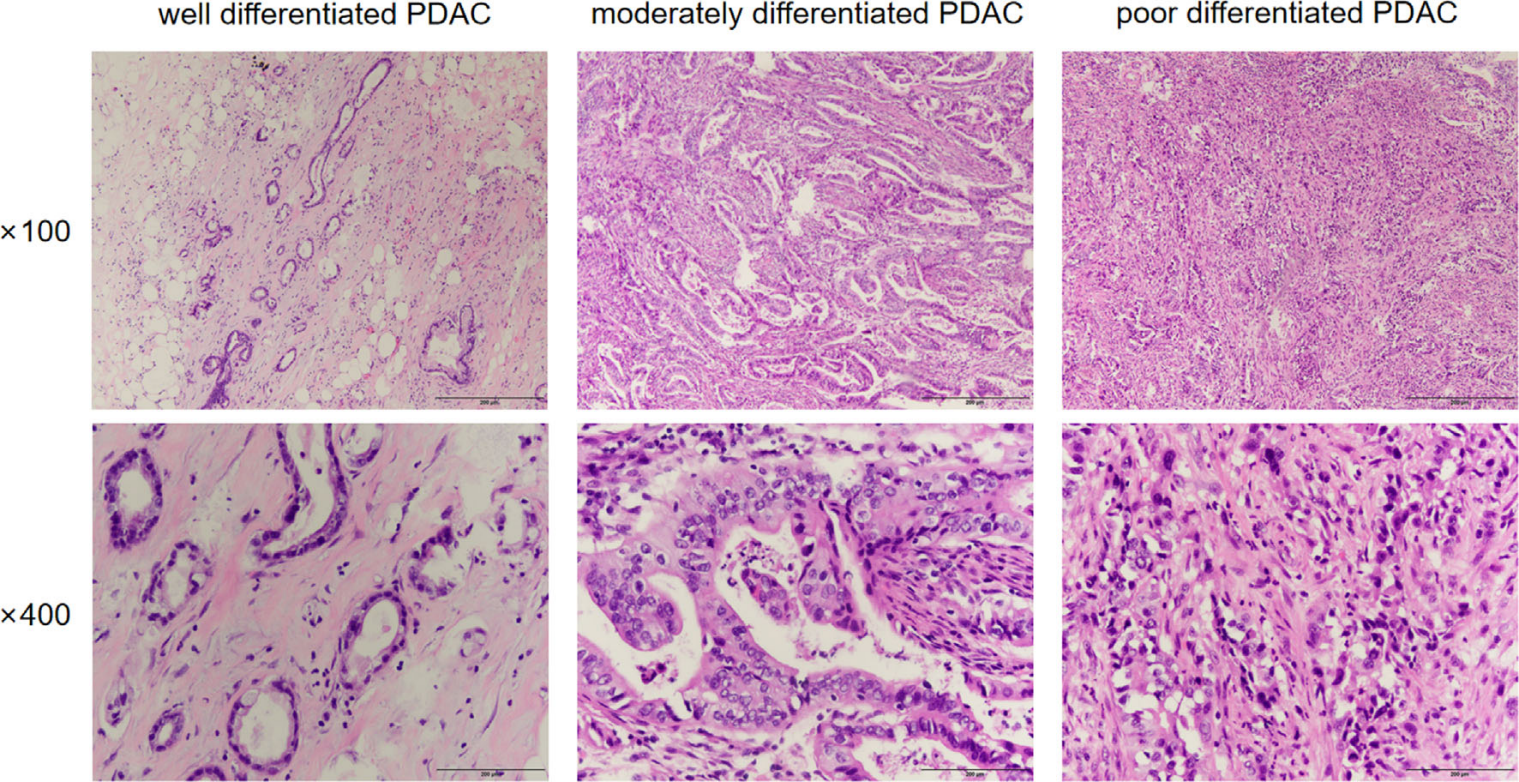
Examples of differentiation at different grades in pancreatic ductal adenocarcinoma (PDAC) stained with H&E.

The pathological diagnosis was established by two professional pathologists. Tumors were staged according to the 8th edition of AJCC TNM staging of pancreatic cancer. Independent of tumor stage, histological tumor grade is an important predictor of disease outcome with higher grade tumors behaving more aggressively. Grade is usually based on microscopic features, including nuclear features. The more closely the tumor resembles normal tissue, the lower the tumor grade and the less aggressive it will behave. Lower‐grade tumors generally grow more slowly and are less likely to spread and metastasize than higher‐grade tumors. The histology grade was dichotomized based on the degree of tumor differentiation: low grade, including well or moderately differentiated adenocarcinoma, and high grade, including poorly differentiated or undifferentiated adenocarcinoma (Figure 1). Lymphovascular infiltration (LVI) refers to the invasion of blood vessels and lymphatic channels, while perineural invasion is the infiltration of peripheral nerves by the primary tumor in close proximity. Patients who received more than three cycles of postoperative adjuvant chemotherapy were classified as the chemotherapy group.

### 2.3 Follow‐up

The patients were reviewed monthly for 6 months following the surgery. Subsequently, they underwent follow‐up evaluations every 3 months for up to 2 years post‐surgery, then every 6 months for up to 5 years, and finally annually or until death. A physical examination, laboratory tests including serum CA19‐9 and CEA levels, and abdominal ultrasound were performed at each visit. Additionally, chest and abdominal computed tomography or abdominal magnetic resonance imaging were routinely obtained every 6 months. Imaging examinations can be performed at any time for patients suspected of recurrence or metastasis. Overall survival (OS) was calculated from the day of surgery until the time of death or final follow‐up, while recurrence‐free survival (RFS) was defined as the interval between surgical time and tumor recurrence or last follow‐up. The median follow‐up was 51 months (range: 1–126) for OS and 49 months (range: 1–126) for RFS, respectively. The date of the final follow‐up was March 30, 2022.

### 2.4 Statistical analysis

The receiver operating characteristic (ROC) curves were used to identify potential cut‐off values of preoperative serum CA19‐9 for predicting survival. The ROC curves were also used to compare the area under the curves (AUC) of the modified TNM staging system and the eighth edition of the TNM staging system, demonstrating their respective advantages in predicting survival. Discriminant function analysis was used to calculate the coefficients for predicting biologic risk based on preoperative serum CA19‐9 levels and histology grade. The OS curves were calculated using the Kaplan–Meier method based on the time elapsed between primary surgical treatment and final follow‐up or death. The log‐rank test was used to assess significant differences between curves. Independent prognostic factors were identified by the COX proportional hazard regression model, which was used to measure homogeneity and discriminatory ability. A bilateral *p*‐value of less than 0.050 was considered statistically significant. The statistical analysis was performed using IBM SPSS 23.0 and MedCalc v.19.6.1.

## 3 RESULTS

### 3.1 Clinicopathological characteristics of the whole cohort

The baseline characteristics of the training cohort are listed in Table 2. The 612 patients included 352 males (57.5%) and 260 females (42.5%). The age ranged from 31 to 82 years, with a median age of 61 years. The median levels of preoperative CA19‐9, CEA, and CA242 were 156.25 U/mL, 3.36 ng/mL, and 19.64 U/mL, respectively. There were 416 patients with PDAC located in the head of the pancreas and 196 patients with PDAC located in the body and tail. A total of 414 patients underwent pancreaticoduodenectomy, 195 underwent distal pancreatectomy, and three underwent total pancreatectomy. Out of the 264 patients with low histology grade, 23 were well differentiated and 241 were moderately differentiated. The remaining 348 poorly differentiated patients were classified as having a high histology grade. The median overall survival (OS) of the training cohort was 18 months (range: 6–126 months), with survival rates of 71.3%, 43.3%, 31.3%, and 19.1% at 1, 2, 3, and 5 years of follow‐up, respectively.

**TABLE 2:** Clinicopathological characteristics of the whole cohort.

Characteristic	Patients(*n* = 612)[%]
Sex	
Male	352[57.5]
Female	260[42.5]
Age median (range)(year)	61(31–82)
CA19‐9 median (range) (U/ml)	156.25(0.6–178850.00)
CEA median (range) (ng/ml)	3.35(0.20–3032.00)
CA242 median (range) (U/ml)	19.64(0.19–11237.00)
Total bilirubin before drainage median (range)(mmol/L)	91.17(3.90–1012.50)
Tumor location	
Head	416[68]
Body and tail	196[32]
Surgery	
Pancreaticoduodenectomy	414[67.6]
Distal pancreatectomy	195[31.9]
Total pancreatectomy	3[0.5]
TNM stage	
I	233[38.1]
II	285[46.6]
III	94[15.4]
T stage	
T1	99[16.2]
T2	323[52.8]
T3	175[28.6]
T4	15[2.5]
N stage	
N0	343[56.0]
N1	191[31.2]
N2	78[12.7]
Histology grade	
Low grade	264[43.1]
Well differentiation	23[3.8]
Moderate differentiation	241[39.4]
High grade	348[56.9]
Poor differentiation	348[56.9]
Undifferentiation	0
Perineural invasion	
No	249[40.7]
Yes	363[59.3]
Lymphovascular invasion	
No	458[74.8]
Yes	154[25.2]
Chemotherapy regime	
None	311[50.8]
GS	80[26.6]
GEMOX	68[22.6]
GEM	61[20.3]
AG	33[11]
S‐1	47[15.6]
FOLFIRINOX	12[4.0]
Overall survival median(range)(months)	18(6–126)

Abbreviations: AG, albumin‐bound paclitaxel and gemcitabine; CA19‐9, carbohydrate antigen 19–9; CA242, carbohydrate antigen 242; CEA, carcinoembryonic antigen; FOLFIRINOX, a combination therapy consisting of 5‐FU/leucovorin plus oxaliplatin and irinotecan; GEM, gemcitabine; GEMOX, gemcitabine and oxaliplatin; GS, gemcitabine and S‐1; TNM stage, the tumor, node and metastasis staging system.

### 3.2 Survival analysis of patients with PDAC

**FIGURE 2:**
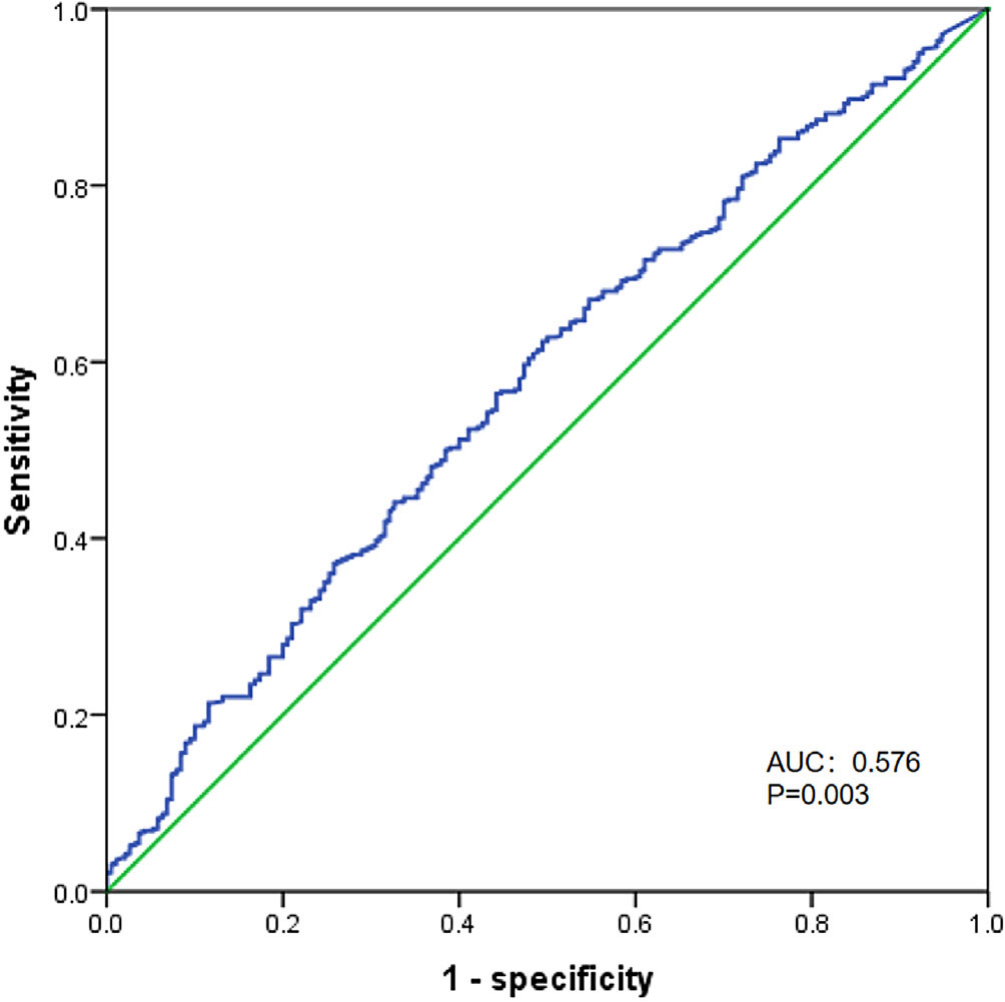
The receiver operating characteristic curve was used to predict the survival of pancreatic cancer based on various cut‐off values of CA19‐9 (AUC = 0.576, 95%CI:0.535–0.615, *p* = 0.003). The optimal cut‐off value for predicting the survival of pancreatic cancer was determined to be 112 U/mL, considering both maximum sensitivity and specificity.

The ROC curves demonstrated that preoperative serum CA19‐9 could predict the prognosis of patients with PDAC (AUC = 0.576, 95% CI: 0.535–0.615, *p* = 0.003) (Figure 2). The optimal cutoff point for preoperative serum CA19‐9 to predict survival was determined to be 112 U/mL. Then, the patients were categorized into two groups based on the optimal cutoff value of preoperative serum CA19‐9: Group 1, in which the level of preoperative serum CA19‐9 was ≤112 U/mL; and Group 2, in which the level of preoperative serum CA19‐9 was >112 U/mL.

The results of the univariate and multivariate survival analyses are presented in Table 3. In the univariate analysis, the following 8 factors were evaluated and found to have a significant effect on survival: age at surgery (<70 years vs. ≥70 years), preoperative CA19‐9 (≤112 U/mL vs. >112 U/mL), preoperative serum CEA (≤5 ng/mL vs. >5 ng/mL), postoperative adjuvant chemotherapy, TNM stage, histology grade (low grade vs. high grade), perineural invasion, and lymphovascular invasion.

**TABLE 3:** Univariate and multivariate survival analysis of all the patients with PDAC.

Characteristics	*n*	5‐year OS(%)	Univariate analysis		Multivariate analysis	
			HR(95% CI)	*p*	HR(95% CI)	*p*
Sex						
Male	352	18.5	1(ref)			
Female	260	18.6	1.118(0.92–1.358)	0.264		
Age at surgery(year)						
<70	525	19.9	1(ref)		1(ref)	
≥70	87	9.2	1.430(1.010–1.857)	0.007	1.546(1.184–2.020)	0.001
CA199 (U/ml)						
≤112	252	22.6	1(ref)		1(ref)	
>112	360	15.9	1.458(1.196–1.778)	<0.001	1.355(1.098–1.671)	0.005
CEA (ng/ml)						
≤5	432	21.7	1(ref)		1(ref)	
>5	180	12.2	1.412(1.146–1.740)	0.001	1.199(0.956–1.504)	0.116
Postoperative chemotherapy						
No	311	14.1	1(ref)		1(ref)	
Yes	301	24.2	0.601(0.496–0.729)	<0.001	0.590(0.485–0.716)	<0.001
TNM stage						
I	233	28.2	1(ref)		1(ref)	
II	285	14.9	1.531(1.235–1.897)	<0.001	1.440(1.153–1.798)	0.001
III	94	7.7	2.279(1.720–3.019)	<0.001	2.140(1.565–2.925)	<0.001
Histology grade						
Low grade	264	23.5	1(ref)		1(ref)	
High grade	348	15.7	1.642(1.348–1.999)	<0.001	1.545(1.265–1.887)	<0.001
Tumor location						
Head	416	19.6	1(ref)			
Body and tail	196	18.2	0.998(0.811–1.227)	0.983		
Perineural invasion						
No	249	25.3	1(ref)		1(ref)	
Yes	363	13.3	1.222(1.004–1.487)	0.046	1.014(0.827–1.243)	0.895
Lymphovascular invasion						
No	458	20.9	1(ref)		1(ref)	
Yes	154	14	1.432(1.155–1.774)	0.001	1.101(0.867–1.397)	0.430

*Note*: Low grade: well and moderate differentiation; high grade: poor differentiation and undifferentiation.

Abbreviations: 95% CI, 95% confidence interval; HR, hazard ratio; OS, overall survival.

**FIGURE 3:**
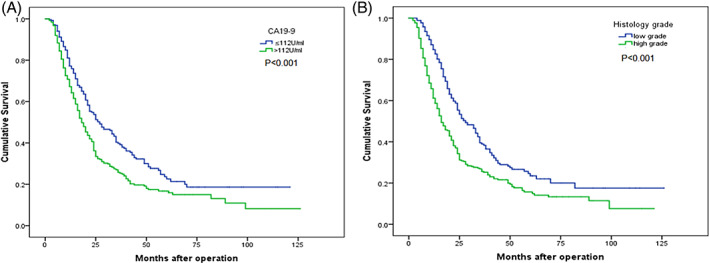
Overall survival curves were analyzed after curative resection. (A) Patients were grouped according to their CA19‐9 levels (*p* < 0.001, log‐rank test). (B) Patients were also grouped based on histology grade (*p* < 0.001), with low grade indicating well and moderate differentiation, and high grade indicating poor differentiation and undifferentiation.

PDAC patients with CA19‐9 levels >112 U/mL had a significantly lower 5‐year overall survival rate than those with CA19‐9 levels ≤112 U/mL (5‐year OS: 15.9% vs. 22.6%, *p* < 0.001, Figure 3A). Additionally, patients with high‐grade tumors also had a significantly lower 5‐year overall survival rate than those with low‐grade tumors (5‐year OS: 15.7% vs. 23.5%, *p* < 0.001, Figure 3B).

In the multivariate analysis, preoperative serum CA19‐9 (with a hazard ratio of 1.355 for CA19‐9 levels >112 U/mL and *p* = 0.005) and histology grade (with a hazard ratio of 1.545 for high grade and *p* < 0.001) were identified as independent prognostic factors for overall survival, along with age at surgery (≥70 years), TNM stage, and postoperative adjuvant chemotherapy.

### 3.3 Development of biological risk model

**FIGURE 4:**
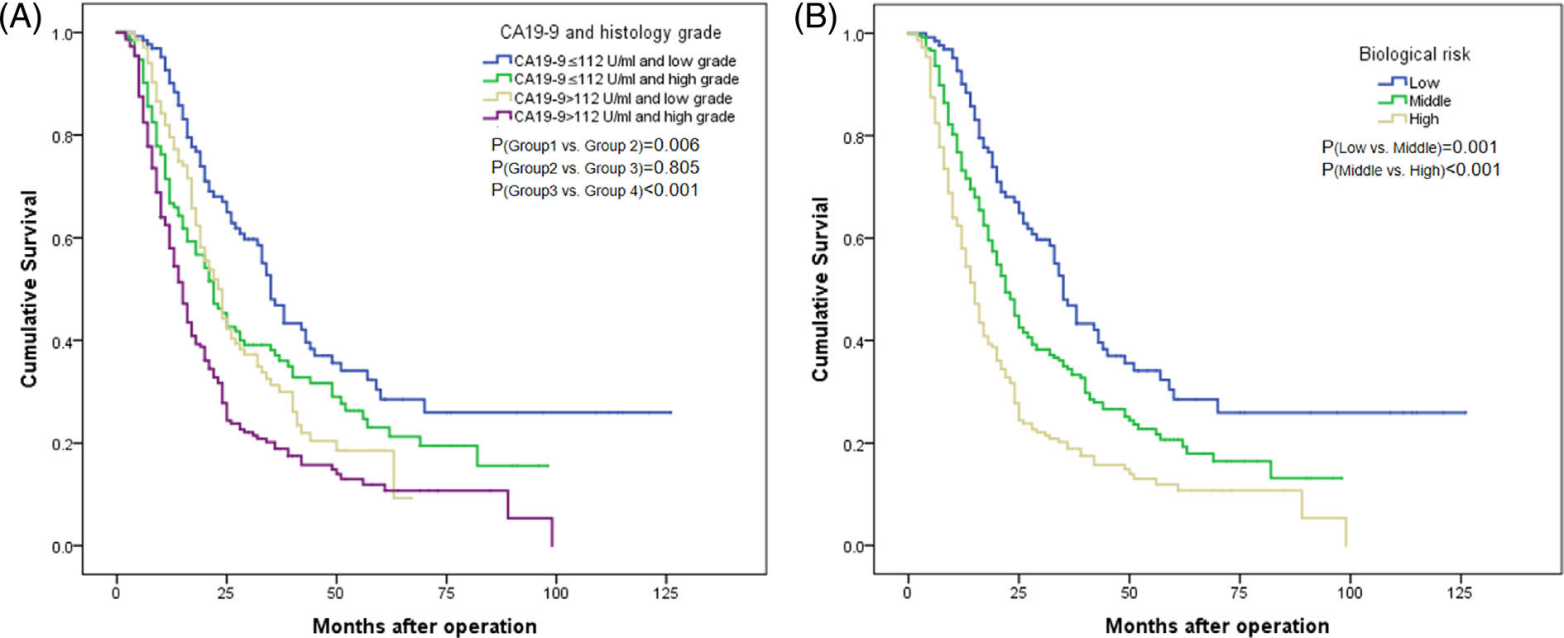
Overall survival curves were plotted after curative resection. (A) The patients were divided into 4 groups based on the status of CA19‐9 and histology grade: group 1 included patients with CA19‐9 ≤ 112 U/mL and low grade; group 2 included patients with CA19‐9 ≤ 112 U/mL and high grade; group 3 included patients with CA19‐9 > 112 U/mL and low grade; group 4 included patients with CA19‐9 > 112 U/mL and high grade. (B) The patients were categorized into 3 groups according to the biological risk: the low‐risk group consisted of patients with CA19‐9 ≤ 112 U/mL and low grade; the middle‐risk group consisted of patients with either CA19‐9 ≤ 112 U/mL + high grade or CA19‐9 > 112 U/mL + low grade; the high‐risk group consisted of patients with both CA19‐9 > 112 U/mL + high‐grade.

Initially, all patients were categorized into four groups based on their preoperative serum CA19‐9 levels and histology grade. Additionally, a survival coefficient was assigned to each group using discriminant function analysis (CA19‐9 × 1.179 + histology grade × 1.600–4.383). Group 1 included 129 patients with CA19‐9 ≤ 112 U/mL and low grade, with a survival coefficient of −1.504; Group 2 had 133 patients with CA19‐9 ≤ 112 U/mL and high grade, with a survival coefficient of −0.004; Group 3 consisted of 134 patients with CA19‐9 > 112 U/mL and low grade, with a survival coefficient of −0.425; and Group 4 comprised 216 patients with CA19‐9 > 112 U/mL and high grade, with a survival coefficient of 1.175. The 5‐year overall survival rates were 28.5%, 23.0%, 18.6%, and 11.9% in Groups 1, 2, 3, and 4, respectively. There was no significant difference in survival between Group 2 and Group 3 (5‐year OS: 23.0% vs. 18.6%, *p* = 0.805) (Figure 4A).

As the survival coefficients of −1 and 1 indicate no difference in survival prediction, we divided the coefficient range into three intervals: <−1, −1 to 1, and >1. Based on these coefficients, a biological risk (BR) model was developed using preoperative serum CA19‐9 levels and histology grade. The low BR group included patients with coefficients <−1; the middle BR group included patients with coefficients between −1 and 1; and the high BR group included patients with coefficients >1. Following this principle, 129 patients with preoperative serum CA19‐9 ≤ 112 U/mL and low grade (coefficients of −1.504) were categorized into the low BR group. Additionally, 267 patients with preoperative CA19‐9 > 112 U/mL and low grade (coefficients of −0.425), as well as those with preoperative serum CA19‐9 ≤ 112 U/mL and high grade (coefficients of −0.004), were categorized into the middle BR group. Finally, 216 patients with preoperative CA19‐9 > 112 U/mL and high grade (coefficients of 1.175) were categorized into the high BR group. The 5‐year OS rates were 28.5%, 14.9% and 7.7% for patients in the low, middle, and high BR groups, respectively (χ^2^ = 92.762, *p* < 0.001) (Figure 4B). In the multivariate analysis, the BR model was found to be an independent prognostic factor for OS (HR was 1.418 for the middle BR group, *p* = 0.016; and HR was 2.221 for the high BR group, *p* < 0.001). (Table 4).

**TABLE 4:** Multivariate survival analyses of all PDAC patients after including biological risk.

Factors	Multivariate analysis		
	HR	95%CI	*p*
Age(year)			
<70	1(ref)		
≥70	1.525	1.169–1.991	0.002
CEA(ng/ml))			
≤5	1(ref)		
>5	1.161	0.928–1.452	0.191
TNM stage			
I	1(ref)		
II	1.456	1.165–1.819	0.001
III	2.164	1.584–2.957	<0.001
Perineural invasion			
No	1(ref)		
Yes	0.993	0.810–1.218	0.950
Lymphvascular invasion			
No	1(ref)		
Yes	1.094	0.861–1.391	0.462
Postoperative chemotherapy			
No	1(ref)		
Yes	0.590	0.485–0.716	<0.001
Biological risk			
Low	1(ref)		
Middle	1.418	1.067–1.886	0.016
High	2.221	1.653–2.983	<0.001

*Note*: Biological risk: Low, CA199 ≤ 112 U/mL + low grade; Middle, CA19‐9 ≤ 112 U/mL + high grade or CA19‐9 > 112 U/mL + low grade; High, CA19‐9 > 112 U/mL + high grade.

### 3.4 Incorporation of biological risk into the eighth edition of the AJCC TNM staging system

**FIGURE 5:**
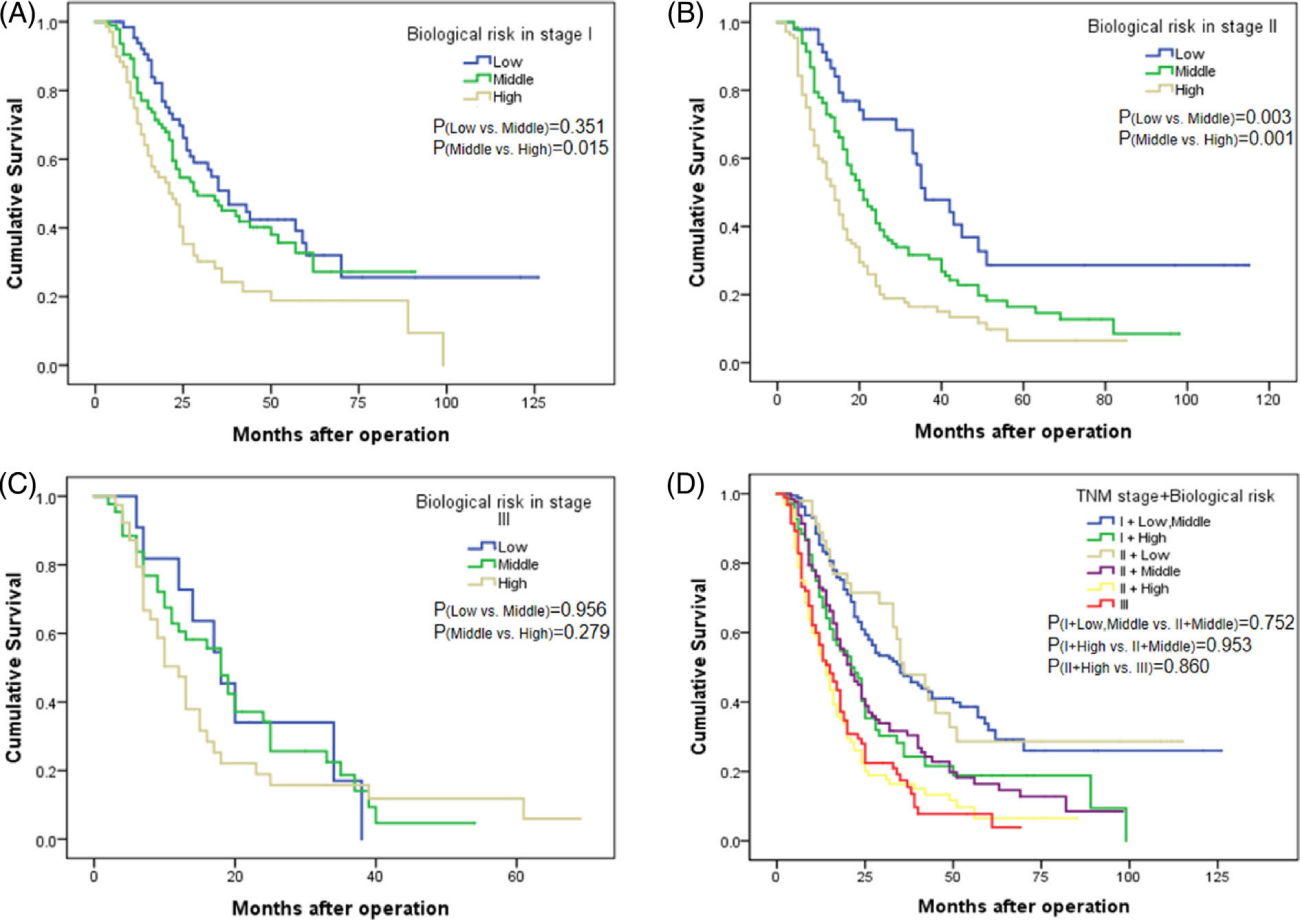
Overall survival curves stratified by TNM stage showed that the survival differences were only observed in patients with stage I and stage II pancreatic cancer. (A) Stage I. (B) Stage II. (C) Stage III. (D) Overall survival curves of pancreatic cancer patients according to TNM stage and biological risk.

With the TNM‐stratified analysis, there was no significant difference in survival between the low BR group and middle BR group in stage I, while the overall survival of these two groups was significantly better than that of the high BR group in stage I (Figure 5A). Among the patients in stage II, there were significant differences in survival between each of the two groups (Figure 5B). There was no significant difference in survival among the three groups of patients in stage III (Figure 5C). The OS of patients in the low BR and middle BR groups, who were at stage I, was similar to that of patients in the low BR group who were at stage II. The OS of patients in the high BR group, who were at stage I, was similar to that of patients in the middle BR group, who were at stage II. The OS of patients in the high BR group, who were at stage II, was similar to that of patients in stage III (Figure 5D).

**FIGURE 6:**
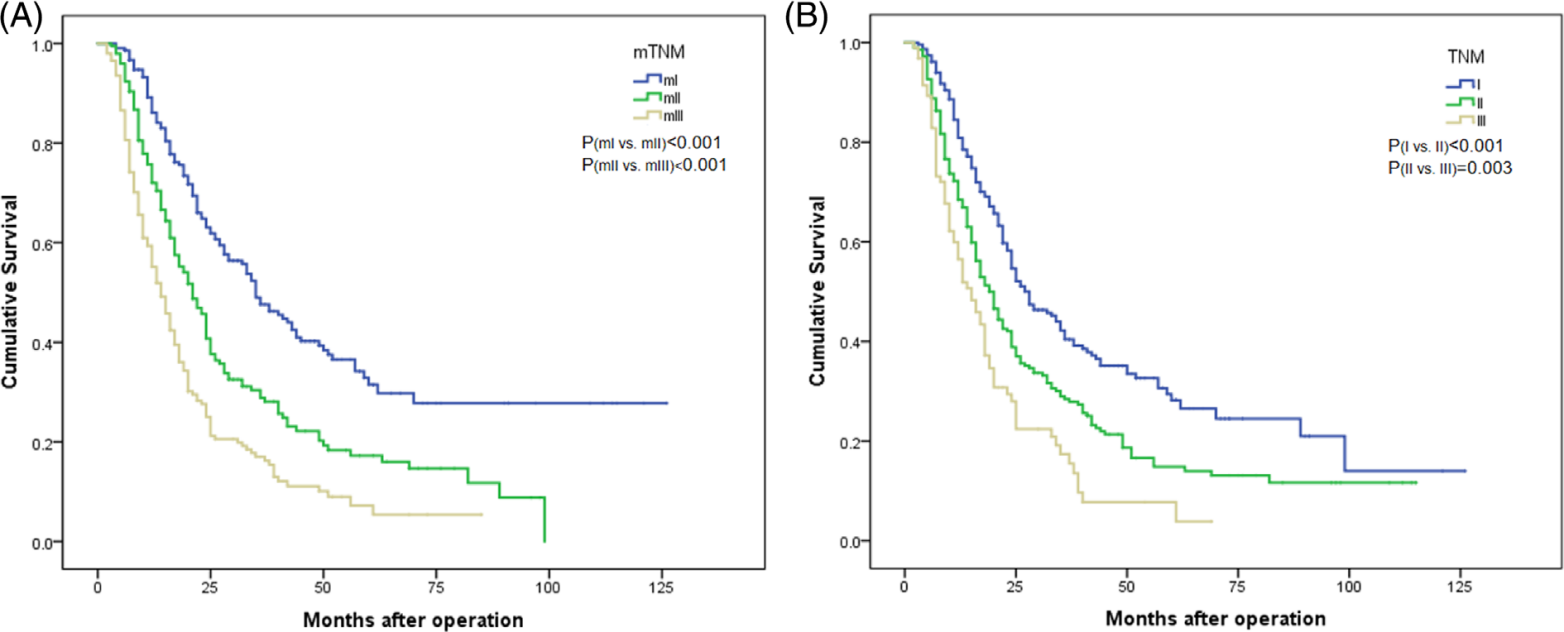
There were significant differences in overall survival (OS) curves between patients with different mTNM or TNM stages of pancreatic cancer in the training cohort. (A) mTNM stage. (B) TNM stage.

Based on the results of the strata analysis, we incorporated BR into the eighth edition of the TNM staging system and introduced our modified TNM (mTNM) staging system (Table 5). In the mTNM staging system, the 5‐year OS rates of stages mI, mII and mIII were 31.5%, 17.2% and 7.2%, respectively (χ^2^ = 78.603, *p* < 0.001). In the TNM staging system, the 5‐year OS rates of stages I, II and III were 28.2%, 14.9% and 7.7%, respectively (χ^2^ = 37.794, *p* < 0.001) (Table 6, Figure 6A,B).

**TABLE 5:** PDAC patients were divided into three groups according to the biological risk of PDAC: Low, CA199 ≤ 112 U/mL + low grade; Middle, CA19‐9 ≤ 112 U/mL + high grade or CA19‐9 > 112 U/mL + low grade; High, CA19‐9 > 112 U/mL + high grade.

Biological risk	I	II	III
Low	mI	mI	mIII
Middle	mI	mII	mIII
High	mII	mIII	mIII

*Note*: The biological risk was incorporated into the eighth edition of TNM staging system and composed the newly modified TNM (mTNM) stage.

**TABLE 6:** The impact of mTNM and TNM on the prognostic value of staging.

Characteristics	*n*	5‐year OS (%)	Univariate analysis		Multivariate analysis		−2log likelihood
			χ^2^	*p*	HR (95% CI)	*p*	
mTNM stage			78.603	<0.001			4688.361
mI	213	31.2			1(ref)	<0.001	
mII	197	17.2			1.627(1.198–2.210)	0.002	
mIII	202	7.2			2.602(1.840–3.678)	<0.001	
TNM stage			37.794	<0.001			4694.876
I	233	28.2			1(ref)	<0.001	
II	285	14.9			1.442(1.155–1.800)	0.001	
III	94	7.7			2.137(1.564–2.921)	<0.001	

Abbreviations: 95% CI, 95% confidence interval; HR, hazard ratio; OS, overall survival.

### 3.5 Predictive performance of the mTNM staging system

**FIGURE 7:**
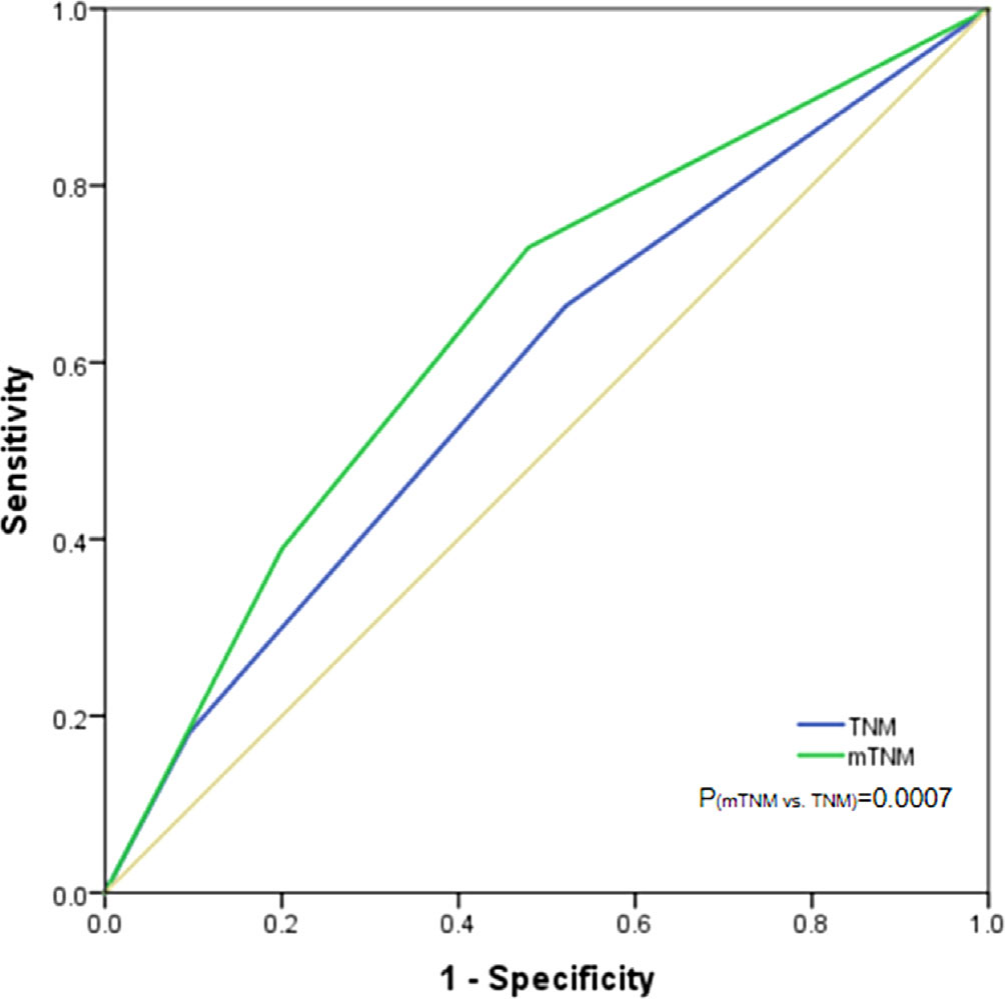
The receiver operating characteristic curve was used to compare the area under curves between mTNM and TNM in the training cohort. AUC(mTNM) was 0.646, AUC(TNM) was 0.587, and *p* value was 0.0007.

The prognostic prediction differences between the eighth edition of the TNM staging system and the mTNM classification system were directly compared. The −2 log likelihood of the mTNM stage was 4688.361, which was lower than that of the TNM staging system (4694.876), indicating a better performance in survival prediction (Table 6). The ROC curves showed that the AUC for mTNM staging was significantly higher than that for TNM staging [AUC: 0.646 (95% CI: 0.606–0.683) vs. 0.587 (0.547–0.626), *p* = 0.0007] (Figure 7).

### 3.6 External validation

**FIGURE 8:**
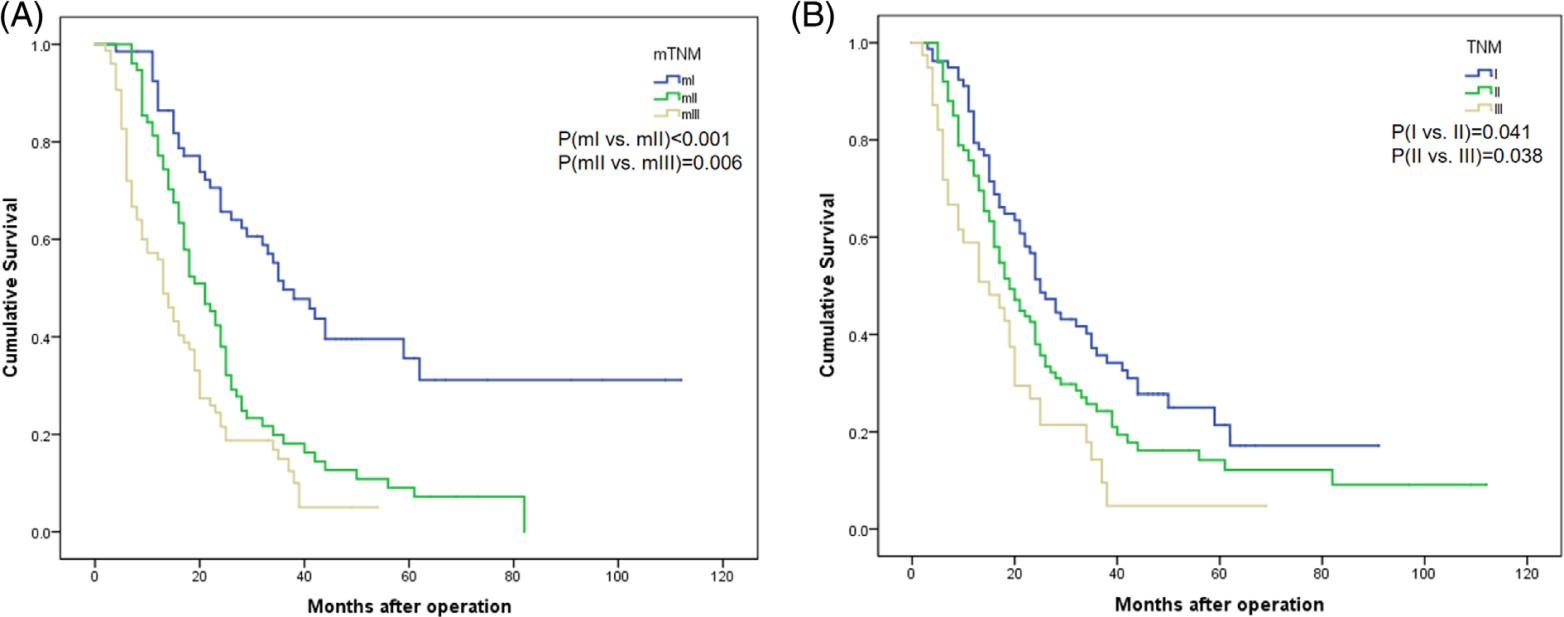
There were significant differences in overall survival (OS) curves between patients with pancreatic cancer in the validation cohort based on their mTNM or TNM stage. (A) mTNM stage. (B) TNM stage.

**FIGURE 9:**
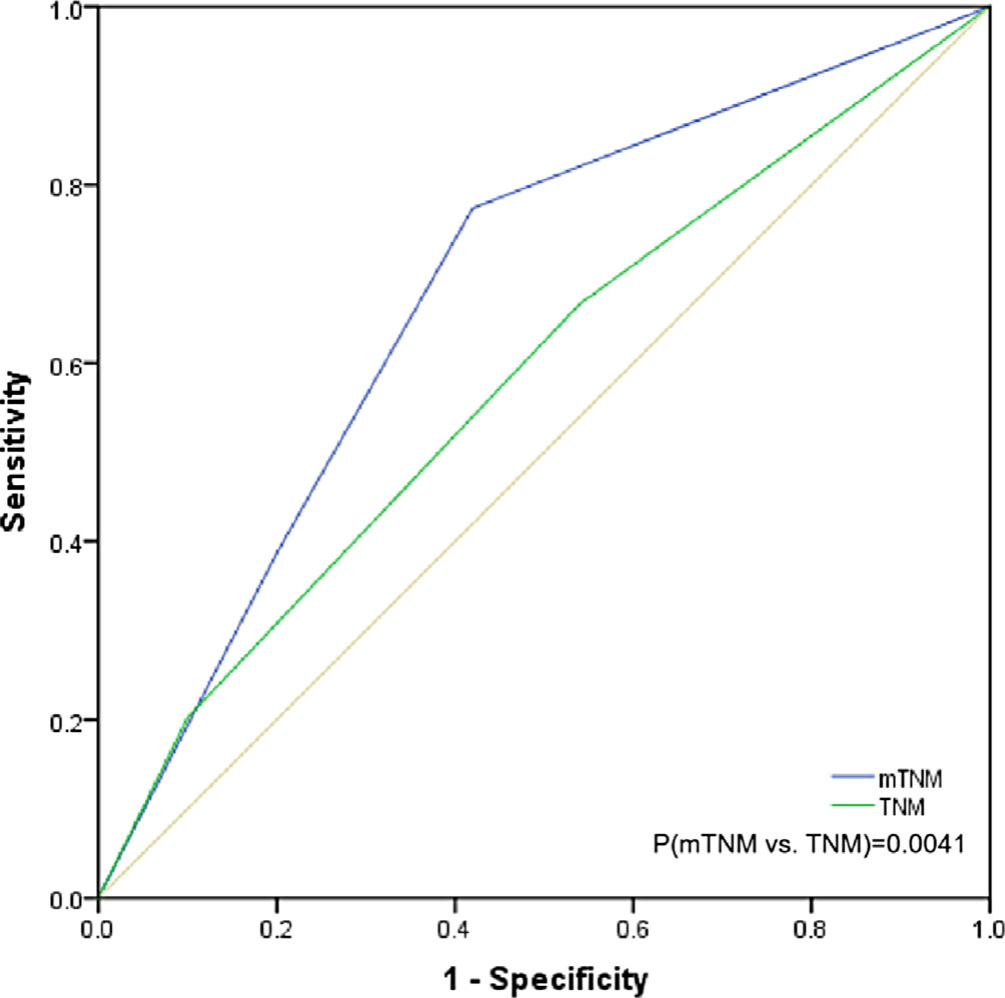
The receiver operating characteristic curve was used to compare the area under curves between mTNM and TNM in the validation cohort. AUC(mTNM) was 0.681, AUC(TNM) was 0.585, with a *p* value of 0.0041.

Ultimately, the proposed staging system underwent external validation. The characteristics of the validation cohort are presented in Table 7. Both the mTNM and TNM staging systems were successfully used to stratify OS into distinct subgroups, as illustrated in Figure 8. However, survival differences between the subgroups of mTNM were more pronounced than those observed with the TNM subgroups. The 5‐year overall survival rates were 35.6%, 9.0%, and 5.0% for stages mI, mII, and mIII, respectively (*p* < 0.001 for mI vs. mII, *p* = 0.006 for mII vs. mIII). The corresponding rates for stages I, II, and III were 21.4%, 14.1%, and 4.8% (*p* = 0.041 for I vs. II, *p* = 0.038 for II vs. III). The mTNM staging system demonstrated superior AUC values compared to the TNM staging system [AUC: 0.681 (95% CI: 0.614–0.742) vs. 0.585 (95% CI: 0.516–0651), *p* = 0.0041)] (Figure 9).

**TABLE 7:** Clinicopathological characteristics of the validation cohort.

Characteristic	Patients(*n* = 218)[%]
Sex	
Male	125[57.3]
Female	93[42.7]
Age median (range)(year)	61(37–80)
CA19‐9 median (range) (U/ml)	195.95(0.19–154370.00)
CEA median (range) (ng/ml)	3.52(0.20–98.58)
CA242 median (range) (U/ml)	23.13(0.33–10342.00)
Total bilirubin before drainage median (range)(mmol/L)	18.20(4.70–833.50)
Tumor location	
Head	164[75.2]
Body and tail	54[24.8]
Surgery	
Pancreaticoduodenectomy	162[74.3]
Distal pancreatectomy	56[25.7]
Total pancreatectomy	0
TNM stage	
I	14[6.4]
II	98[45.0]
III	106[48.6]
Histology grade	
Low grade	91[41.7]
Well differentiation	5[2.3]
Moderate differentiation	86[39.4]
High grade	127[58.3]
Poor differentiation	127[58.3]
Undifferentiation	0
Perineural invasion	
No	87[39.9]
Yes	131[60.1]
Lymphovascular invasion	
No	161[73.9]
Yes	57[26.1]
Chemotherapy regime	
None	59[28.9]
GS	47[23.0]
GEMOX	65[31.8]
AG	12[5.9]
S‐1	14[6.9]
FOLFIRINOX	7[3.4]
Overall survival median (range) (months)	20(3–80)

Abbreviations: AG, albumin‐bound paclitaxel and gemcitabine; CA19‐9, carbohydrate antigen 19–9; CA242, carbohydrate antigen 242; CEA, carcinoembryonic antigen; FOLFIRINOX, a combination therapy consisting of 5‐FU/leucovorin plus oxaliplatin and irinotecan; GEM, gemcitabine; GEMOX, gemcitabine and oxaliplatin; GS, gemcitabine and S‐1; TNM stage, the tumor, node and metastasis staging system.

## 4 DISCUSSION

Both tumor burden and malignant degree can reflect the biological characteristics of a tumor, and they are also associated with the survival of patients with malignancy. The AJCC TNM classification, which reflects tumor burden, is the most widely used staging system for malignancy. However, it is a macroscopic classification mainly based on anatomy and does not incorporate factors that reflect microscopic tumor burden (which cannot be detected by imaging),^16^ nor does it consider factors that reflect the malignant degree of a tumor. As a result, there are always accuracy biases when using the TNM stage to predict prognosis.^10^, ^11^ In the present study, we aimed to incorporate preoperative serum CA19‐9, which could compensate for the deficiency of TNM stage in evaluating microscopic tumor burden, and histology grade, which could make up for the defect of TNM stage in reflecting malignant degree, into the TNM staging system to reduce biases.

In this study, patients with a preoperative serum CA19‐9 level > 112 U/mL or high histology tumor grade demonstrated a significantly lower overall survival rate than those with CA19‐9 ≤ 112 U/mL or low histology tumor grade (5‐year OS: 15.9% vs. 22.6%, *p* < 0.001; 15.7% vs. 23.5%, *p* < 0.001). Both preoperative serum CA19‐9 and histology grade were identified as independent prognostic factors for overall survival in the multivariate analysis. Our proposed biological risk (BR) based on preoperative serum CA19‐9 and histological grade is an independent prognostic factor for PDAC. After incorporating the BR model into the eighth edition of the TNM staging system, we established the mTNM staging system, which was confirmed to be more accurate in predicting postoperative prognosis for PDAC patients than the eighth edition of the AJCC TNM staging system.

CA19‐9 is the most widely used serum tumor marker. Kang et al. reported that both pretreatment and post‐treatment levels of CA19‐9, as well as their changes after treatment, have good prognostic value in determining the survival of pancreatic cancer patients.^19^ Currently, there is no established cutoff value for CA19‐9 to predict the prognosis of PDAC^20^, ^21^, ^22^ as its levels fluctuate widely. In our study, CA19‐9 levels ranged from 0.6 to 178 850 U/mL. Additionally, the proportion of Lewis antigen‐negative patients varies across countries, ethnicities and studies.^23^, ^24^, ^25^, ^26^ However, multiple studies have confirmed the superiority of CA19‐9 over other serum tumor markers in predicting the prognosis of PDAC^27^, ^28^, ^29^ which is consistent with our findings.

Histology grade is defined as the level of differentiation exhibited by a tumor, which refers to the degree of morphological and functional similarity between a tumor cell and a normal cell from the same tissue. Malignant neoplasms typically progress from low grade to high grade. During the process of malignancy, tumors become more aggressive, and the tumor burden increases as the tumor grade rises.^30^, ^31^ Our study also found that histology grade was significantly associated with the survival of patients with PDAC and could be used to modify the TNM staging system as demonstrated in many previous studies.^17^, ^18^

To the best of our knowledge, this study is the first to categorize patients with PDAC according to their biological risk based on preoperative CA19‐9 levels and histology grade and assess the impact of tumor biology on survival. In our opinion, the combination of preoperative serum CA19‐9 levels and histology grade provides unique prognostic information that reflects microscopic tumor burden and malignancy degree, which are not included in the TNM staging system. Incorporating biological risk into the TNM staging system may classify patients with the same TNM stage into different subgroups, and it may also facilitate the design of individualized treatment regimens.

There are several limitations to the study. First, it was a retrospective study conducted at a single center. Second, patients included in the study were from 2011 to 2018; therefore, current neoadjuvant therapy was not routinely used. Additionally, we could not exclude patients who were negative for Lewis antigen, which may have an uncertain impact on accuracy. As the constraints listed above may introduce bias in the results, further validation is necessary through large‐scale and prospective multicenter studies to confirm its superiority and applicability.

## 5 CONCLUSION

Overall, biological risk based on preoperative serum CA19‐9 and histological grade can be used to predict the survival of patients with PDAC. By incorporating this biological risk into the TNM staging system, the accuracy of patient classification can be improved, thus facilitating the development of individualized treatment plans.

## AUTHOR CONTRIBUTIONS

All authors had full access to the data in the study and take responsibility for the integrity of the data and the accuracy of the data analysis. **Methodology:** Shaofei Chang and Yaohua Liu; **Data Curation:** Shaofei Chang; **Formal Analysis:** Shaofei Chang and Yaohua Liu; **Writing–Original Draft:** Shaofei Chang and Yaohua Liu; **Investigation:** Yaohua Liu and Quan Man; **Software:** Haorui Li and Yu Guo; **Writing–Review & Editing:** Tiansuo Zhao and Shaofei Chang; **Project Administration:** Tiansuo Zhao.

## CONFLICT OF INTEREST STATEMENT

The authors have stated explicitly that there are no conflicts of interest in connection with this article.

## ETHICS STATEMENT

This study was approved by the Ethical Review Committees of Tianjin Medical University Cancer Institute and Hospital. Written informed consent was obtained from all patients before surgery that included a statement regarding the collection of clinicopathological data and samples for scientific purposes.

## Data Availability

The data that support the findings of this study are available from the corresponding author upon reasonable request.
